# Thermal and efficiency droop in InGaN/GaN light-emitting diodes: decoupling multiphysics effects using temperature-dependent RF measurements

**DOI:** 10.1038/s41598-019-56390-2

**Published:** 2019-12-27

**Authors:** Arman Rashidi, Morteza Monavarian, Andrew Aragon, Daniel Feezell

**Affiliations:** Center for High Technology Materials (CHTM), University of New Mexico, Albuquerque, New Mexico 87106 USA

**Keywords:** Lasers, LEDs and light sources, Inorganic LEDs

## Abstract

Multiphysics processes such as recombination dynamics in the active region, carrier injection and transport, and internal heating may contribute to thermal and efficiency droop in InGaN/GaN light-emitting diodes (LEDs). However, an unambiguous methodology and characterization technique to decouple these processes under electrical injection and determine their individual roles in droop phenomena is lacking. In this work, we investigate thermal and efficiency droop in electrically injected single-quantum-well InGaN/GaN LEDs by decoupling the inherent radiative efficiency, injection efficiency, carrier transport, and thermal effects using a comprehensive rate equation approach and a temperature-dependent pulsed-RF measurement technique. Determination of the inherent recombination rates in the quantum well confirms efficiency droop at high current densities is caused by a combination of strong non-radiative recombination (with temperature dependence consistent with indirect Auger) and saturation of the radiative rate. The overall reduction of efficiency at elevated temperatures (thermal droop) results from carriers shifting from the radiative process to the non-radiative processes. The rate equation approach and temperature-dependent pulsed-RF measurement technique unambiguously gives access to the true recombination dynamics in the QW and is a useful methodology to study efficiency issues in III-nitride LEDs.

## Introduction

III-nitride light-emitting diodes (LEDs) emitting in the ultraviolet (UV) and visible spectrum have broad applications in solid-state lighting (SSL) systems, biosensing, visible-light communication, and emissive micro-LED displays^1^. Despite decades of development, III-nitride LEDs still suffer from efficiency issues such as *efficiency droop*^2,3^, *thermal droop*^4^, the *green gap*^5^, and efficiency in UV LEDs remains low^6^. Among these issues, thermal and efficiency droop in blue InGaN/GaN LEDs are more studied due to their limiting role in achieving efficient SSL systems and reducing the global energy consumption and lumen per area cost of the LED chips. Based on more than a decade of studies, many carrier mechanisms have been proposed as the potential cause of droop phenomena. Theories concerning the origin of droop at high carrier densities and elevated temperatures can be organized into two main categories: (1) reduction of the radiative efficiency^7–11^ and (2) current injection loss due to carrier leakage from the active region^12–16^.

An ideal study of thermal and efficiency droop should simultaneously consider the effects of both radiative efficiency reduction and current injection efficiency. The radiative efficiency is an inherent property of the active region and depends on the recombination dynamics while the injection efficiency is the fraction of injected current that is consumed by recombination process in the active region and depends on both active and cladding region designs. A common method to study the recombination dynamics in the active region of LEDs is to measure the radiative efficiency and carrier recombination lifetime and decouple the radiative and non-radiative recombination rates in the quantum-wells (QWs). However, many previous carrier recombination lifetime studies were performed with optically pumped techniques, which do not capture the effects of carrier dynamics under typical electrically injected operating conditions, giving an incomplete picture of the physics behind droop phenomena^17,18^. On the contrary, under electrically injected RF methods carriers experience carrier-carrier interaction between the QWs and cladding regions, electrical injection effects are captured into the carrier lifetime measurement, and a flat-band is achieved. However, previous RF methods used to obtain the carrier recombination lifetime under electrical injection do not decouple the injection and carrier transport effects from the inherent carrier recombination in the QW^7,19–21^. Recently, we used a rate equation approach and RF measurement technique to decouple the injection and transport effects from the inherent carrier dynamics in the QW, resulting in the extraction of the true differential carrier lifetime (DLT)^22,23^. Moreover, previous RF measurements were performed under continuous-wave (CW) operation, where heat generated in the active region is not independently controlled, affecting the carrier dynamics of the LED and obscuring the fundamental recombination processes. Finally, most electrically injected techniques are unable to separate the injection efficiency from the inherent radiative efficiency, undermining the role of carrier leakage and ballistic overshoot. Thus, decoupling of the inherent radiative efficiency, injection efficiency, carrier recombination lifetime, carrier transport, and thermal effects under electrical injection is essential to create a complete picture of the physics associated with thermal and efficiency droop.

Recently, we have reported on a comprehensive rate equation approach and electrically injected RF measurement technique to extract the carrier recombination lifetime and injection efficiency in III-nitride LEDs^22,23^. Here, we build upon this method to extract the inherent radiative and non-radiative recombination rates in the InGaN QW of semipolar ($20\overline{2}\overline{1}$) LEDs by decoupling the injection and carrier transport effects from the carrier dynamics in the QW. We also remove the thermal effects on carrier dynamics using a technique that enables measurement of the RF characteristics of the LEDs under pulsed operating conditions. Rate equations are used to formulate the time dependence of the various carrier mechanisms in the LED and derive an equivalent electrical circuit representing these carrier mechanisms. Simultaneous fitting of the modulation response and input impedance of the circuit to the measured modulation response and input impedance of the LED gives lifetimes for the various carrier mechanisms in the LED, extracting parameters such as the injection efficiency, carrier transport, carrier density, and total recombination rate in the QW. Finally, we determine the inherent radiative and non-radiative recombination rates in the QW by coupling the total recombination rate in the QW with the true radiative efficiency of the LED. The radiative efficiency was found by knowing the injection efficiency extracted from the RF measurement and the internal quantum efficiency (IQE) of the LED. The studies were carried out at different stage temperatures and various bias current densities to target thermal and efficiency droop, respectively. The rate equation approach and pulsed-RF measurement technique yields the inherent radiative and non-radiative recombination rates by excluding the injection, transport, and thermal effects from the carrier dynamics in the QW of InGaN/GaN LEDs.

## Results

### Carrier dynamics in InGaN/GaN LEDs and derivation of equivalent electrical circuit

**Figure 1:**
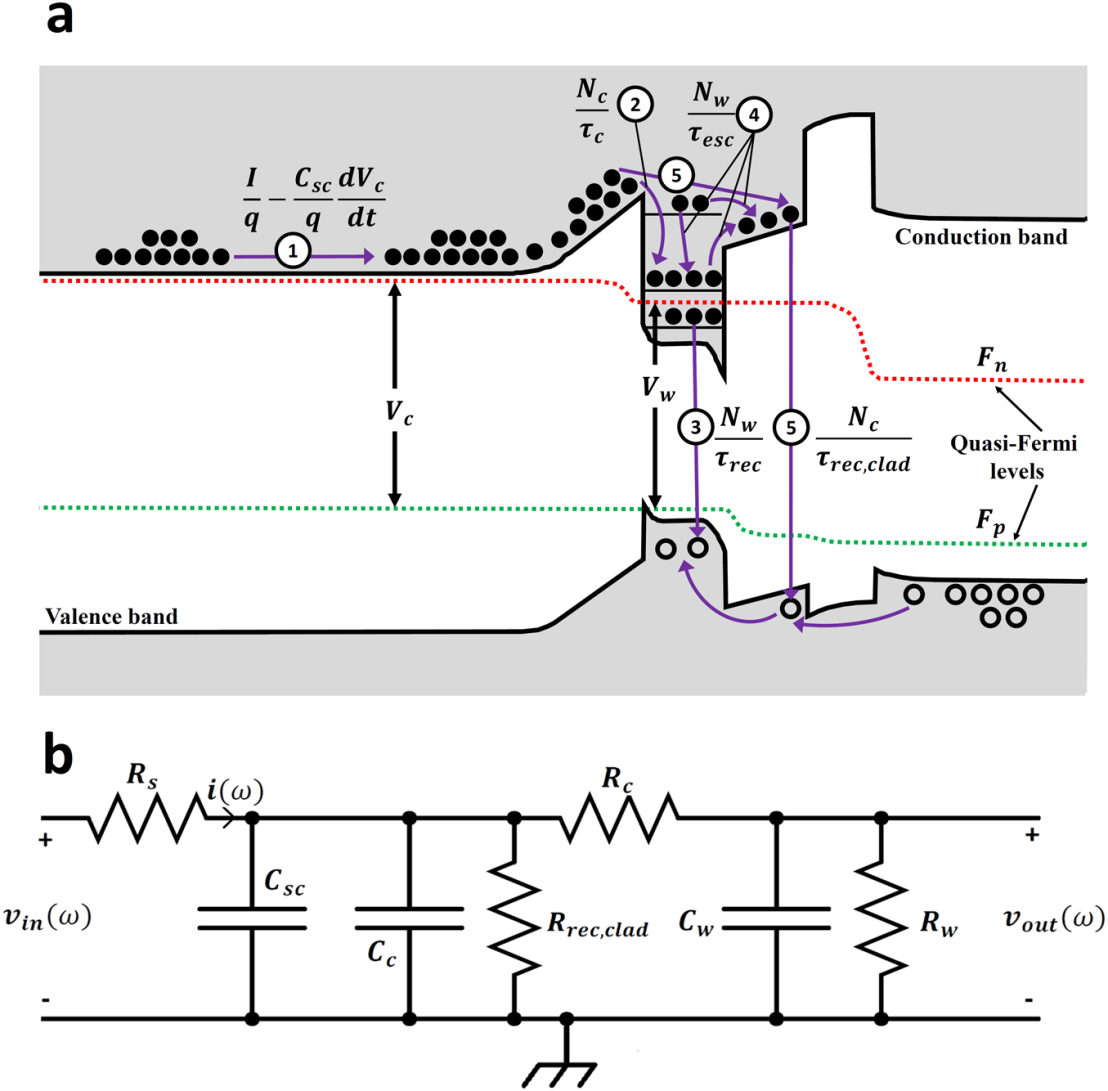
(**a**) Energy band diagram of the LED under flat-band conditions at a current density of 1 kA/cm^2^ simulated using SiLENSe. The original bandgap is reduced to show a more detailed picture of the bandgap and quasi-fermi levels. Dominant carrier processes with their associated rates are shown. (**b**) Equivalent electrical circuit of the LED.

Carrier rate equations were formulated considering the energy band diagram and dominant carrier mechanisms shown in Fig. 1(a). We write the single-particle rate equations by assuming the carrier dynamics are mostly governed by electrons (e.g., hole leakage is negligible). In our previous work, we showed that the excellent agreement of our model with experimental data confirms the accuracy of this assumption^23^. In Fig. 1(a), *N*_*c*_ is the number of unconfined carriers in the cladding regions. *N*_*w*_ is the number of confined and unconfined carriers inside and above the QW. *V*_*c*_ and *V*_*w*_ are the separation of the quasi-fermi levels in the cladding layers and QW, respectively. The various processes are numbered in Fig. 1(a). 1: *I* is the current injected into the LED and ${C}_{sc}\frac{d{V}_{c}}{dt}$ is the current required to charge the space-charge capacitance of the junction (*C*_*sc*_). 2: Carriers in the cladding layer (*N*_*c*_) diffuse toward (*τ*_*diff*_) and are captured by (*τ*_*cap*_) the QW with a rate of $\frac{{N}_{c}}{{\tau }_{c}}$, where ${\tau }_{c}={\tau }_{diff}+{\tau }_{cap}$ is the total delay that carriers experience in the cladding layer. 3: Confined carriers in the QW can either recombine within the QW (radiatively or non-radiatively) with a total rate of $\frac{{N}_{w}}{{\tau }_{rec}}$ or 4: escape the QW by thermionic emission (*τ*_*th*_). The unconfined carriers above the QW either overflow (*τ*_*of*_) to the cladding regions or are captured by the QW through Coulomb-enhanced capture (*τ*_*cou*_). Coulomb-enhanced capture is an interband carrier-carrier scattering process where an unconfined carrier is captured by the QW due to a coulombic force with a carrier in the cladding^19^. This process is dominant at high current densities where the population of carriers in the QW and cladding is similar. Therefore, the net carrier escape rate is $\frac{{N}_{w}}{{\tau }_{esc}}=\frac{{N}_{w}}{{\tau }_{th}}+\frac{{N}_{w}}{{\tau }_{of}}-\frac{{N}_{w}}{{\tau }_{cou}}$. 5: Unconfined carriers in the cladding layers recombine with a rate of $\frac{{N}_{c}}{{\tau }_{rec,clad}}$, where *τ*_*rec*_,_*clad*_ is the carrier recombination lifetime in the claddings. High energy carriers that are ballistically transported to the opposite side most likely recombine along with the escaped carriers from the QW as minority carriers and their effect is folded into *τ*_*rec*_,_*clad*_. Some of the escaped or ballistically transported carriers are re-captured by the QW and their effect is embedded in *τ*_*cap*_. Therefore, the rate equations for the cladding regions and the QW are as follows:

1 $$\frac{d{N}_{c}}{dt}=\frac{I}{q}-\frac{{C}_{sc}}{q}\frac{d{V}_{c}}{dt}-\frac{{N}_{c}}{{\tau }_{c}}+\frac{{N}_{w}}{{\tau }_{esc}}-\frac{{N}_{c}}{{\tau }_{rec,clad}}$$

2 $$\frac{d{N}_{w}}{dt}=\frac{{N}_{c}}{{\tau }_{c}}-\frac{{N}_{w}}{{\tau }_{rec}}-\frac{{N}_{w}}{{\tau }_{esc}}.$$

The differential forms of Eqs. (1) and (2) lead to the extraction of the equivalent electrical circuit of the LED shown in Fig. 1(b). *R*_*w*_ (*R*_*C*_) and *C*_*w*_ (*C*_*C*_) are the resistance and capacitance associated with carriers in the QW (cladding), respectively. ${R}_{rec,clad}$ is the resistance associated with carriers recombining in the cladding region. Resistances and capacitances are related to different lifetimes through ${\tau }_{rec}={R}_{w}{C}_{w}$, ${\tau }_{c}={R}_{c}{C}_{c}$, ${\tau }_{rec,clad}={R}_{rec,cald}{C}_{c}$, and ${\tau }_{esc}={R}_{c}{C}_{w}$.

*R*_*s*_ is the parasitic series resistance of the LED added to the intrinsic equivalent circuit manually. Details of the extraction of the circuit and its elements can be found elsewhere^22,23^. Values of the resistances and capacitances are reported in Supplementary Section 6. The differential rate equations are also used to derive expressions for the injection efficiency and carrier density in the QW (see Supplementary Section 3). Simultaneous fitting of the measured modulation response and input impedance of the LED to the theoretical modulation response and input impedance of the circuit of Fig. 1(b) enables the extraction of the differential form of carrier lifetimes in Eqs. (1) and (2). The injection efficiency and carrier density in the QW are then calculated using the extracted lifetimes.

### Pulsed-RF measurement

**Figure 2:**
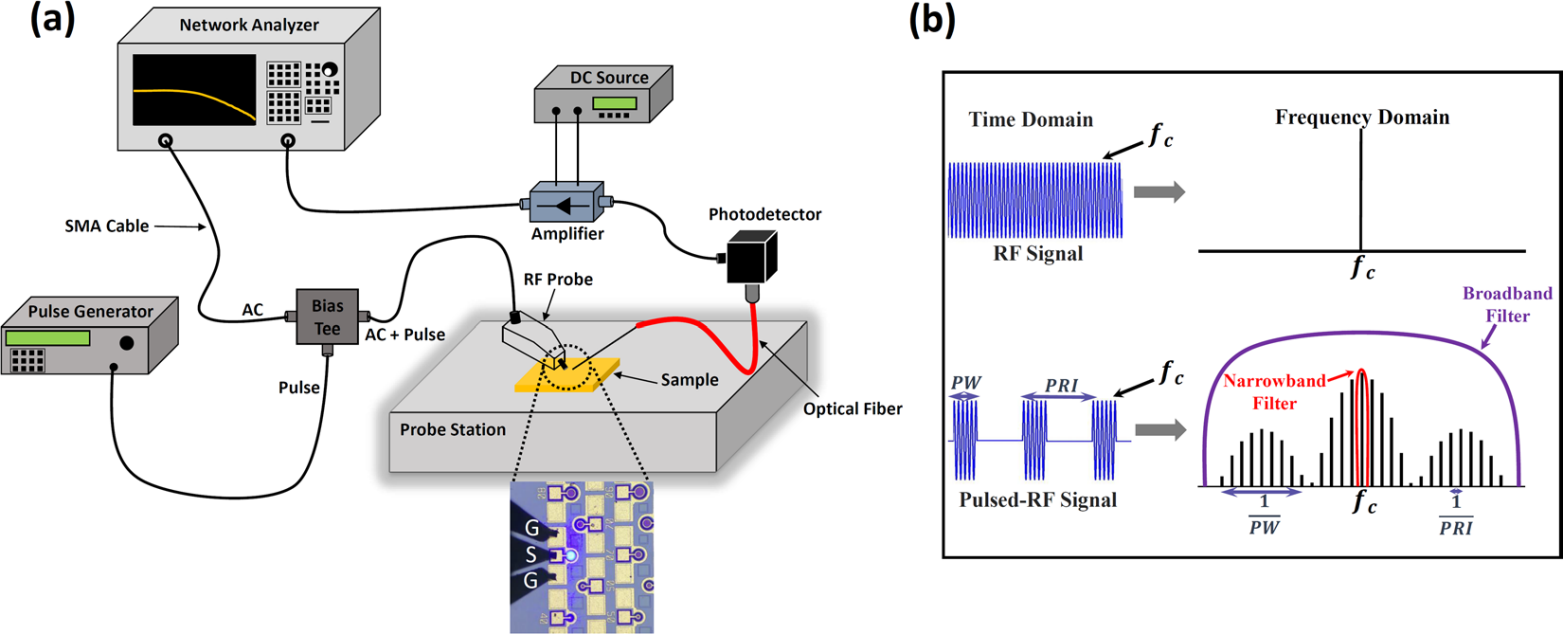
(**a**) RF setup used to measure the modulation response and input impedance of the LED. The optical microscope image of the LED under RF probe is included. (**b**) Concept of the pulsed-RF measurement developed to minimize the effect of self-heating on the measurements. A narrowband filter of the NA is used to pick the main harmonic of the RF frequency among all the generated harmonics.

To measure the modulation response and input impedance under isothermal conditions and exclude self-heating effects from the carrier dynamics, the LED was biased by DC pulses from a pulse generator (AVTECH 1010B) with a pulse repetition interval (PRI) of 10 µsec and a pulse width (PW) of 1 µsec (measurement of the LED output power as a function of current density for different pulse duty cycles confirmed negligible self-heating in LEDs under this pulse condition). Then, a small AC signal from the network analyzer (NA) was added to the DC pulses and sent to the LED. The S-parameters of the LED were measured using the RF setup shown in Fig. 2(a). The received signals by the NA are in the form of pulsed-RF signals due to the LED being off during the off-state of the pulse. Figure 2(b) shows the transmitted (RF) and received (pulsed-RF) signals by the NA both in time and frequency domains. Unlike the continuous-wave (CW) mode where the NA receives signals with the same frequency (*f*_*c*_) as the signal sent to the LED, in pulsed mode the energy of the signal is spread out into infinite harmonics of the main frequency (*f*_*c*_). To receive the main signal back, a very broadband receiver (few hundred MHz) is needed to collect all the harmonics, which interfere constructively and generate the main signal. Such a broadband receiver still does not exist in state-of-the-art NAs. However, here, a narrowband filter (NA’s internal hardware) was used to pick only the central harmonic of the signal among all the generated harmonics (see Fig. 2(b)), since it has the same frequency as the input signal. Although most of the energy of the signal is lost to the remaining harmonics, the main frequency can be detected similarly to the CW mode, enabling S-parameter evaluation by the NA.

### Efficiency and RF characteristics of InGaN/GaN LEDs

**Figure 3:**
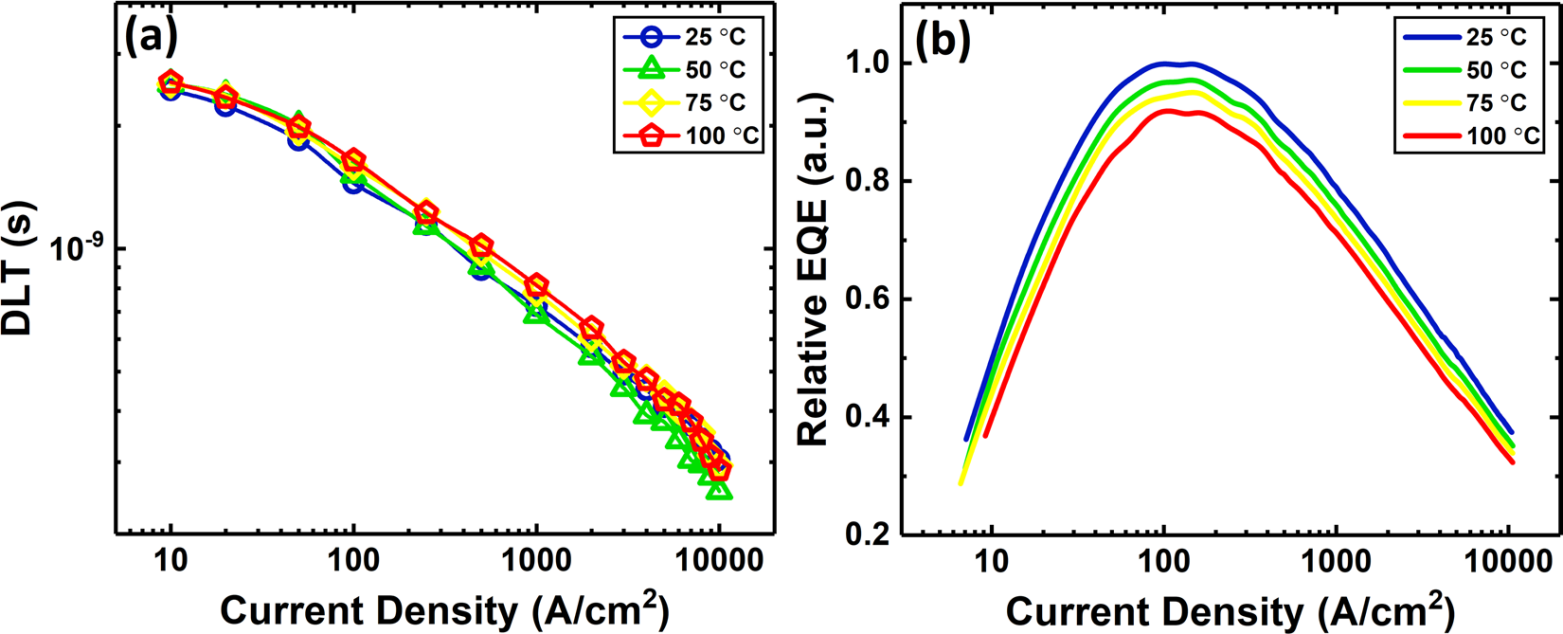
(**a**) The DLT extracted from simultaneous fitting of the measured input impedance and modulation response to the input impedance and modulation response of the equivalent circuit of the LED (Fig. 1(b)) and (**b**) relative EQE of the LED as a function of current density at different stage temperatures.

The modulation response and input impedance of the LED were measured using the pulsed-RF technique at current densities ranging from 10 A/cm^2^ to 10 kA/cm^2^ and at different stage temperatures ranging from 25 °C to 100 °C. The differential carrier lifetime (DLT), net differential carrier escape time, and differential recombination lifetime and transport time in the cladding regions were extracted as a function of current density and stage temperature after simultaneous fit of the input impedance and modulation response of the circuit in Fig. 1(b) to the measured input impedance and modulation response of the LED for the frequency range of 10 MHz to 3 GHz. Details of the circuit derivation and fittings are reported elsewhere and in Supplementary Section 4 ^22–24^. Figure 3(a) shows the DLT (*τ*_Δ*rec*_) as a function of current density at different stage temperatures. The DLT reduces with increasing current density but is almost independent of temperature. David *et al*.^19^ reported a similarly weak temperature dependence for the DLT obtained by a small-signal RF approach. The inconsistency in the DLT at high current density for a temperature of 50 °C is due to slight measurement variation which mathematically propagates into other parameters derived based on the DLT such as the total carrier lifetime and radiative and non-radiative lifetimes and rates. Similar to the RF measurements, the relative external quantum efficiency (EQE) of the LED was measured under the same pulsed conditions at the same current densities and stage temperatures (see Fig. 3(b)). The EQE shows a hot/cold factor ($\frac{{{\rm{EQE}}}_{100{}^{\circ }{\rm{C}}}}{{{\rm{EQE}}}_{25{}^{\circ }{\rm{C}}}}$) of 80–90%, which is similar to previously reported semipolar $(20\overline{2}\overline{1})$ LEDs with similar epi structures^25,26^.

## Discussion

**Figure 4:**
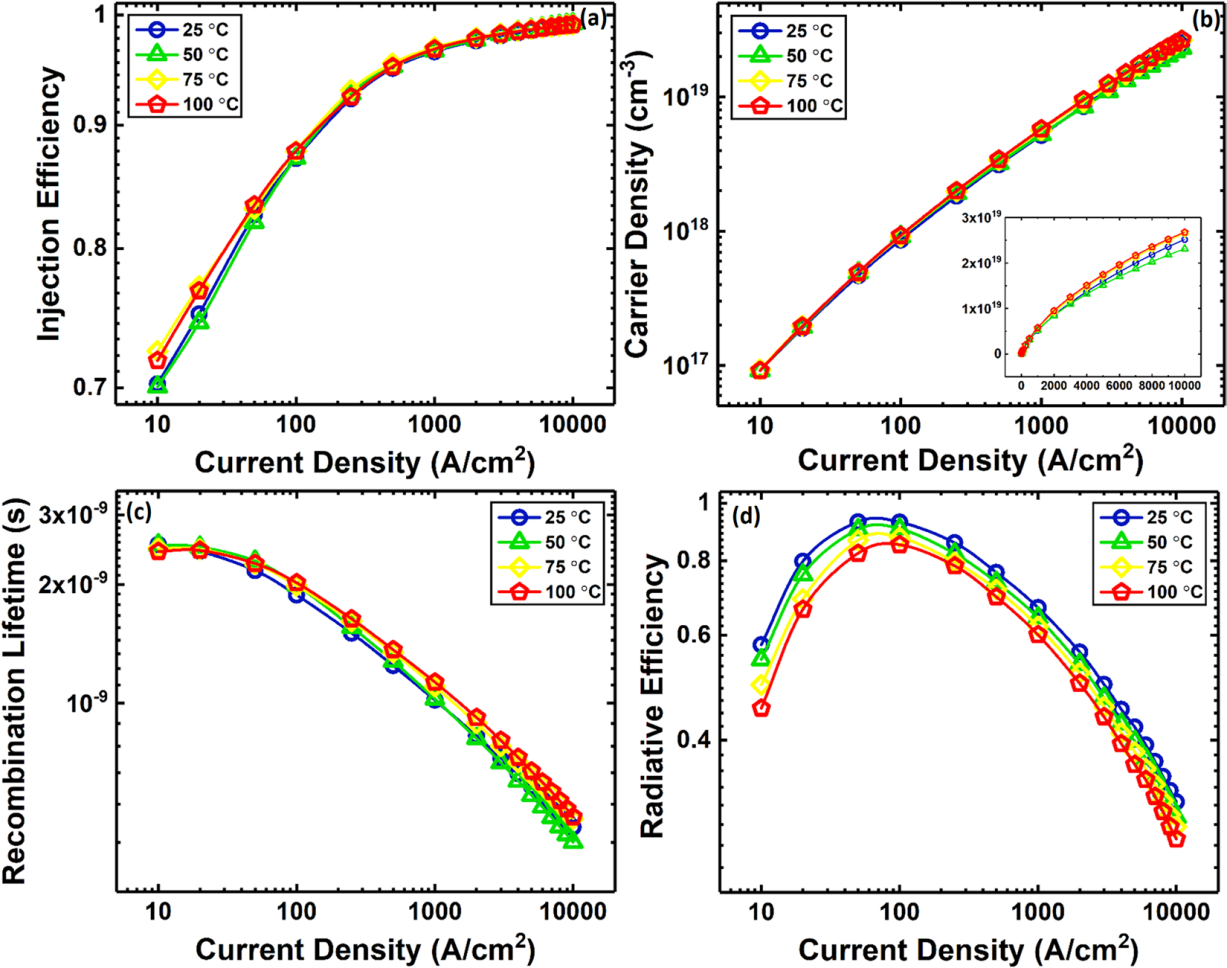
(**a**) Injection efficiency, (**b**) carrier density in the QW, (**c**) total carrier recombination lifetime in the QW, and (**d**) radiative efficiency as a function of current density at different stage temperatures. The inset of (**b**) shows the carrier density *vs*. current density in linear-linear scale.

The injection efficiency and carrier density in the QW were calculated using the extracted lifetimes and are shown in Fig. 4(a,b), respectively. A detailed discussion of the behavior of the injection efficiency and carrier density can be found in our previous work and Supplementary Section 3^22^. The injection efficiency at all temperatures increases with current density and eventually approaches one. Thus, the injection efficiency cannot account for either efficiency droop or thermal droop. The weak temperature dependence of the injection efficiency also confirms that higher temperatures do not lead to stronger carrier leakage from the QW, but rather increase the carrier participation in the non-radiative processes. Carrier escape time as a function of current density for different stage temperatures is also shown in Supplementary Section 5. Knowing the DLT and carrier density in the QW (*n*_*w*_), the total carrier recombination rate (*R*_*rec*_) and lifetime (*τ*_*rec*_) in the QW are calculated as follows^27^:

3 $${R}_{rec}={\int }_{0}^{{n}_{w}}\frac{d{n}_{w}}{{\tau }_{\Delta rec}}$$

4 $${\tau }_{rec}=\frac{{n}_{w}}{{R}_{rec}}$$

To calculate the integral of Eq. (3), the DLT at a current density of zero is needed. We estimated the DLT at a current density of zero to be 2.6, 2.5, 2.4, and 2.3 ns for temperatures of 25, 50, 75, and 100 °C, respectively, by extrapolating the DLT curve at low current densities to zero current density^7,21^. Figure 4(c) shows the calculated total carrier recombination lifetime. The total carrier recombination lifetime is analogous to the carrier lifetime obtained by the time-resolved photoluminescence (TRPL) technique^28^. The radiative efficiency (*η*_*r*_) in Fig. 4(d) was extracted using the measured relative EQE (*η*_*EQE*_) of Fig. 3(b) and the injection efficiency (*η*_*inj*_) in Fig. 4(a) by assuming a constant extraction efficiency (*η*_*ext*_) and using ${\eta }_{EQE}={\eta }_{ext}{\eta }_{inj}{\eta }_{r}$. This procedure results in the relative radiative efficiency. To adjust the relative *η*_*r*_ curves to absolute values, we set the peak of the *η*_*r*_ data at a temperature of 25 °C to 93%, which was previously measured for this sample using room-temperature/low-temperature PL techniques^29^. Because the PL measurement uses resonant optical pumping, which generates carriers directly in the QW, the injection efficiency is ~100%. Therefore, the peak IQE obtained using the PL method is equal to the peak radiative efficiency of the QW. The rest of the curves were calculated relative to the *η*_*r*_ at 25 °C. Note that Fig. 4(d) shows the inherent radiative efficiency, with the injection, carrier transport, and thermal effects decoupled.

The weak temperature dependence of the carrier recombination lifetime and the presence of thermal droop in the radiative efficiency data implies that at elevated temperatures carriers in the QW only exchange their loss channel from the radiative process to the non-radiative processes (e.g., Shockley-Read-Hall (SRH) and Auger), resulting in thermal droop (since radiative lifetime is equal to $\,\frac{{\tau }_{rec}\,}{{\eta }_{r}}$, decrease of *η*_*r*_ (Fig. 4(d)) and constant *τ*_*rec*_ (Fig. 4(c)) with increasing temperature requires radiative lifetime to increase which implies non-radiative lifetime must decrease to maintain a constant DLT). In other words, if a simple ABC model is assumed, with increasing temperature the A and C coefficients increase while the B coefficient decreases, resulting in a constant DLT and reduction of efficiency.

**Figure 5:**
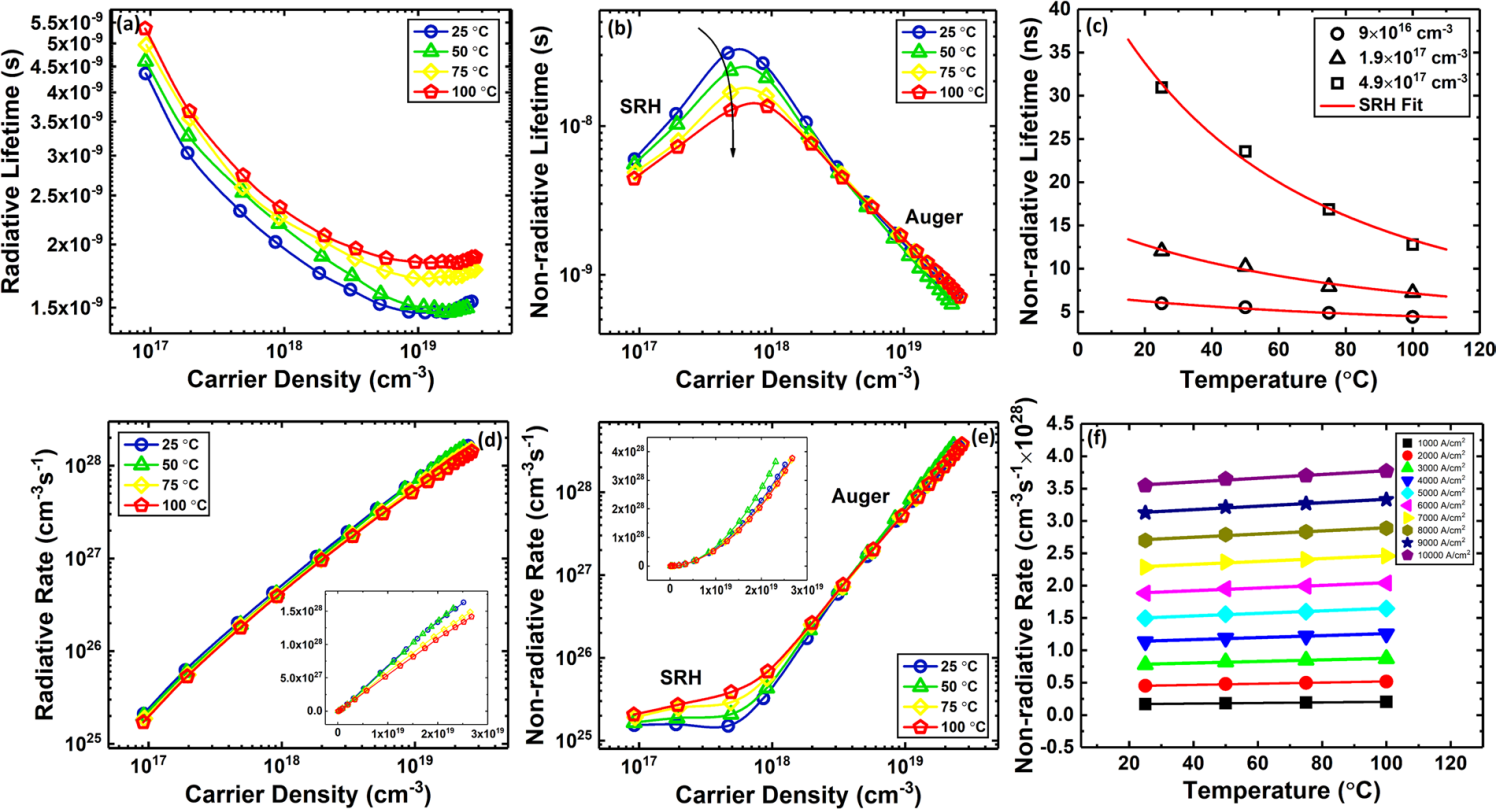
(**a**) Radiative and (**b**) non-radiative lifetimes, (**c**) is the non-radiative lifetime at carrier densities of 9 × 10^16^, 1.9 × 10^17^, and 4.9 × 10^17^ cm^−3^ and the fitting of Eq. (5) for various stage temperatures. (**d**) Radiative and (**e**) non-radiative recombination rates as a function of carrier density for different stage temperatures. The low slope of the non-radiative rate as a function of carrier density at low carrier densities is due to SRH recombination while the high slope at carrier densities above 10^18^ cm^−3^ is attributed to Auger recombination. The inset of (**d**,**e**) show the radiative and non-radiative rates *vs*. carrier density in linear-linear scale. (**f**) Non-radiative recombination rate as a function of temperature for current densities of 1 to 10 kA/cm^2^.

With *η*_*r*_, carrier density, and the carrier recombination lifetime (*τ*_*rec*_) known from Fig. 4, the radiative (*τ*_*r*_) and non-radiative (*τ*_*nr*_) lifetimes in the QW were extracted (The radiative efficiency and carrier recombination lifetime are related to the radiative and non-radiative lifetimes through ${{\rm{\eta }}}_{{\rm{r}}}=\frac{{{\rm{\tau }}}_{{\rm{nr}}}}{{{\rm{\tau }}}_{{\rm{nr}}}+{{\rm{\tau }}}_{{\rm{r}}}}$ and ${{\rm{\tau }}}_{{\rm{rec}}}=\frac{{{\rm{\tau }}}_{{\rm{nr}}}{{\rm{\tau }}}_{{\rm{r}}}}{{{\rm{\tau }}}_{{\rm{nr}}}+{{\rm{\tau }}}_{{\rm{r}}}}$, respectively). Figure 5(a,b) show the radiative and non-radiative lifetimes at various carrier densities for different stage temperatures. The non-radiative lifetime compared to the radiative lifetime decreases drastically with increasing carrier density, reducing the radiative efficiency at high carrier densities. A recent theoretical calculation of radiative and Auger lifetimes vs. carrier density shows similar dependencies, where the Auger lifetime has a stronger dependence on carrier density compared to the radiative lifetime^30^. These behaviors are expected from the ABC model by simply comparing the radiative and Auger lifetimes, ${\tau }_{r}=\frac{1}{Bn}$ vs. ${\tau }_{Auger}=\frac{1}{C{n}^{2}}$^31,32^. Based on the phase space filling theory^33,34^, at high carrier densities *Bn* approximately has a constant behavior as a function of *n* ($Bn\propto {B}_{0}$), while *Cn*^2^ is a linear function of *n* ($C{n}^{2}\propto {C}_{0}n$). According to Fig. 5(a), the onset of phase-space filling for the radiative lifetime occurs at a carrier density of 5 × 10^18^, which is similar to that reported by David *et al*.^7^ Phase-space filling also reduces the slope of the non-radiative lifetime at high injection levels in Fig. 5(b) with increasing carrier density.

At higher temperatures, the radiative lifetime increases at all carrier densities while the non-radiative lifetime decreases significantly only at low carrier densities (<2 × 10^18^ cm^−3^) and is less dependent on temperature at high carrier densities. We attribute the temperature-dependent non-radiative lifetime at low carrier densities to SRH recombination. The SRH lifetime (*τ*_*SRH*_) is related to temperature through^35^:

5 $${\tau }_{SRH}={\tau }_{0}(1+cosh\frac{{E}_{T}-{E}_{Fi}}{kT})$$

where *τ*_0_ is a constant related to trap densities, *E*_*T*_ is the energy of the traps, and *E*_*Fi*_ is the intrinsic fermi level. The initial increase of the non-radiative lifetime with increasing carrier density is attributed to the saturation of SRH recombination centers and has been previously observed in InGaN/GaN LEDs^36,37^. Shockley *et al*.^38^ in their initial publication on statistics of recombination showed that recombination through traps is dependent on carrier density (*n*) (e.g. ${\tau }_{SRH}={\tau }_{p0}+{\tau }_{n0}(\frac{n}{n+{n}_{0}})$ where *τ*_*p*0_ and *τ*_*n*0_ are carrier lifetimes and *n*_0_ is background carrier density). We observed a similar peak in the non-radiative lifetime as a function of carrier density for the semipolar LEDs studied here extracted using photoluminescence (PL) and time-resolved PL (TRPL) measurements^21,29^. Although the peak in the non-radiative lifetime is relatively significant in the semipolar LED, it is not always observed in *c*-plane LEDs^7^. In the case of some *c*-plane QWs, the initial shorter non-radiative lifetime at low carrier densities may be artificially lengthened due to poor electron-hole wavefunction overlap as a result of polarization-related electric fields, causing the peak in the non-radiative recombination lifetime of Fig. 5(b) to vanish.

According to Fig. 5(b) with increasing temperature, the SRH lifetime decreases and its initial rise with increasing carrier density reduces. The initial increase (positive slope at low temperatures) of the non-radiative lifetime with increasing carrier density confirms that the non-radiative lifetime at low carrier densities is dominated by SRH recombination since Auger-dominated recombination would exhibit a negative slope with increasing carrier density. Thus, we estimate the non-radiative lifetime at the lowest carrier density (9 × 10^16^ cm^−3^) to be the SRH lifetime by assuming Auger recombination is negligible^7^. Note from Fig. 4(d) that these LEDs do not show droop until >10^18^ cm^−3^. Figure 5(c) shows the non-radiative lifetime at carrier densities of 9 × 10^16^, 1.9 × 10^17^, and 4.9 × 10^17^ cm^−3^ and the theoretical fitting of Eq. (5) as a function of temperature. At a carrier density of 9 × 10^16^ cm^−3^, the fitting parameters are *τ*_0_ = 1.2 *ns* and ${E}_{T}-{E}_{fi}=53\,meV$.

The SRH lifetimes at a current density of 9 × 10^16^ cm^−3^ range from 6 ns to 4.4 ns at temperatures of 25 °C and 100 °C, respectively. The trends of the SRH lifetime as a function of temperature are similar to those obtained from optical measurements and reported by Nguyen *et al*.^39^ and Meyaard *et al*.^40^

Figure 5(d,e) show the total radiative (*R*_*r*_) and non-radiative (*R*_*nr*_) recombination rates in the QW calculated using Eqs. (6) and (7).

6 $${R}_{r}=\frac{{n}_{w}}{{\tau }_{r}}$$

7 $${R}_{nr}=\frac{{n}_{w}}{{\tau }_{nr}}.$$

Both rates increase with increasing current density, with the non-radiative rate having a higher slope and the radiative rate tending to saturate at high current densities. The radiative and non-radiative rates determine the radiative efficiency of the LEDs through ${\eta }_{r}=\frac{{R}_{r}}{{R}_{r}+{R}_{nr}}$. Figure 5(d,e) show that a combination of a higher non-radiative recombination rate and the saturation of the radiative rate leads to efficiency droop in these LEDs. The non-radiative rate as a function of carrier density has two distinctive slopes, the low slope at low carrier densities (<10^18^ cm^−3^) is due to SRH recombination while the high slope at carrier densities above 10^18^ cm^−3^ is attributed to Auger recombination. Moreover, the radiative rate reduces with temperature at all carrier densities, while the non-radiative rate has a strong temperature dependence at low carrier densities but weaker dependence at high current densities. The stronger temperature dependence of the non-radiative rate at low carrier density is attributed to SRH recombination^39,40^, while the weaker dependence on temperature at high carrier densities is consistent with indirect Auger recombination.

Figure 5(f) shows the non-radiative rate as a function of temperature for current densities ranging from 1 to 10 kA/cm^2^. The non-radiative rate is a linear function of temperature for these current densities. The trend of the non-radiative rate as a function of temperature at high current densities is dictated by the trend of carrier density *vs*. temperature (Fig. 4(b)), while the non-radiative lifetime does not exhibit a clear trend *vs*. temperature (see Eq. 7 and Fig. 5(b)) at high carrier densities. Kioupakis *et al*.^10,41^ theoretically showed that the Auger process in nitride LEDs is dominated by indirect scattering that has a weaker dependence on temperature (monotonically increasing) compared to direct Auger processes (exponential)^42^. They show that over a similar temperature range there is an insignificance increase in the computed *C* coefficient^41^. The trend of non-radiative rate at high current densities in Fig. 5(f) is consistent with indirect Auger process. Kioupakis *et al*. also theoretically calculated the radiative (*B*) and Auger (*C*) coefficients as a function of temperature and observed that *B* decreases at elevated temperatures while *C* increases (similar to Fig. 5(d,f), respectively)^43^. They suggested that the temperature dependence of *C* is consistent with the indirect Auger process. Moreover, experimental work has shown similar temperature dependence for radiative and Auger recombination^4,44^. Our observations are consistent with these reports, concluding that thermal droop is caused by a decrease of the radiative rate and an increase of SRH and Auger recombination with increasing temperature. Higher temperatures cause carriers in the QW to shift from the radiative process to the non-radiative processes. Therefore, thermal and efficiency droop are not the same phenomenon; however, they are both caused by recombination dynamics in the QW.

## Conclusions

In conclusion, we have extracted the inherent radiative and non-radiative recombination rates, carrier density, and injection efficiency as a function of current density and temperature in electrically injected SQW InGaN/GaN LEDs using a comprehensive rate equation approach and a pulsed-RF measurement technique. The rate equation approach and pulsed-RF measurement technique yields the true radiative and no-radiative recombination rates in the QW of InGaN/GaN LEDs by decoupling the injection, transport, and thermal effects from the carrier dynamics in the active region. The trends of injection efficiency as a function of current density and temperature exclude injection efficiency as the primary cause of efficiency and thermal droop. Furthermore, the strong non-radiative recombination rate and saturation of the radiative rate at high current densities are responsible for efficiency droop. The reduction of the radiative rate and increase of the non-radiative rate at elevated temperatures show that carriers move from the radiative process to the non-radiative processes with increasing temperature, leading to thermal droop. The temperature dependence of the non-radiative rate was consistent with SRH and indirect Auger recombination at low and high current densities, respectively. This work experimentally confirms previous first-principles theoretical work and elucidates the individual roles of different carrier mechanisms in thermal and efficiency droop in III-nitride LEDs. The rate equation approach and temperature-dependent pulsed-RF measurement technique is useful for the study of efficiency issues in III-nitride LEDs.

## Methods

### LED structure

High-brightness semipolar ($20\overline{2}\overline{1}$) LEDs with a single-quantum-well (SQW) active region were grown on a free-standing GaN substrate using metal organic chemical vapor deposition (MOCVD). LEDs grown on semipolar ($20\overline{2}\overline{1}$) exhibit low efficiency droop, high brightness, large optical polarization, narrow emission linewidth, high indium incorporation efficiency, and robust temperature stability, making the semipolar ($20\overline{2}\overline{1}$) LEDs good candidates for studies of droop^21,25,26,45–50^. The SQW active region was chosen to avoid carrier transport effects present in multi-quantum-well (MQW) LEDs. We have applied this method to SQW and MQW LEDs on *c*-plane and *m*-plane substrates and observed similar overall behaviors. Injection efficiency of LEDs on different orientation of GaN are shown in Supplementary Fig. S1. The LED structure includes a 2-µm-thick *n*-GaN, 12-nm-thick unintentionally doped (UID) GaN barrier, 12-nm-thick In_0.15_Ga_0.85_N single QW, 12-nm-thick UID GaN barrier, 10-nm-thick *p*-Al_0.20_Ga_0.80_N electron blocking layer (EBL), 100-nm-thick *p*-GaN, and a 10-nm-thick *p*++-GaN. Micro-LEDs with RF-compatible electrodes were fabricated to accommodate the high-speed RF probe. A device with 50-µm-diameter circular mesa was studied in this paper. The fabrication details and Electroluminescence (EL) spectrum of the LED are discussed in Supplementary Section 2. The growth and electrical characterization details of the device are reported elsewhere^21,29^.

## Supplementary Information

Supplementary Information

## Data Availability

The datasets generated during and/or analyzed during the current study are available from the corresponding author on reasonable request.
