# Supplementary material for: Thermal and efficiency droop in InGaN/GaN light-emitting diodes: decoupling multiphysics effects using temperature-dependent RF measurements
Source: Sci Rep. 2019 Dec 27;9:19921. doi: 10.1038/s41598-019-56390-2 (PMC6934866; doi:10.1038/s41598-019-56390-2)
Supplement: Supplementary file 1 — Supplementary Information [file 41598_2019_56390_MOESM1_ESM.pdf]

Supplementary Information for

Thermal and efficiency droop in InGaN/GaN light-emitting diodes: decoupling multiphysics effects using temperature-dependent RF measurements

Arman Rashidi*, Morteza Monavarian, Andrew Aragon, and Daniel Feezell

*Corresponding Author: arashidi@unm.edu

Center for High Technology Materials (CHTM), University of New Mexico, Albuquerque, New Mexico, 87106, USA

1. Injection efficiency of LEDs grown on different orientations of GaN

We have observed similar injection efficiency trends for MQW *c*-plane (0001) and *m*-plane $(10\bar{1}0)$ LEDs, suggesting that the higher injection efficiency with increasing current density is not strongly orientation dependent. Figure S1 shows the injection efficiency as a function of current density for polar, semipolar, and nonpolar LEDs. The injection efficiency at low current densities

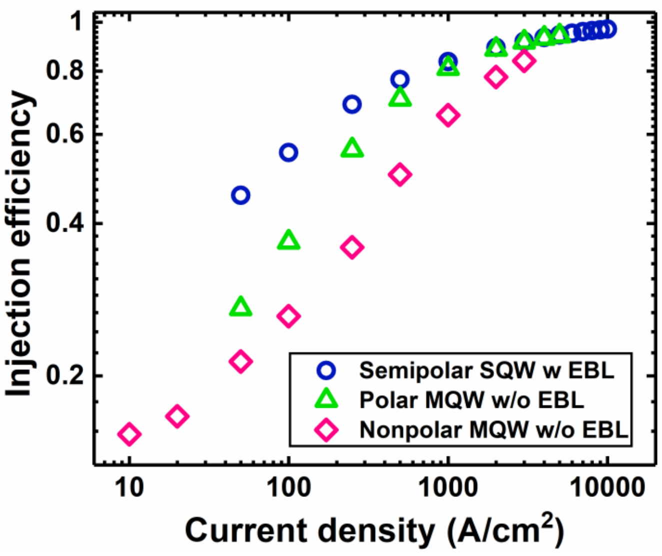

Figure S1 The injection efficiency for MQW polar, semipolar, and nonpolar LEDs as a function of current density.

depends on epitaxial structure and material properties of the LED. For instance, the injection efficiency of the semipolar LED is the highest due to the presence of an electron blocking layer (EBL), which reduces the electron leakage. The injection efficiency of the nonpolar LED is lower than that of the polar LED because of the lower acceptor doping in the *p*-GaN layer of the nonpolar LED, leading to lower hole injection and consequently higher electron leakage.

2. Micro-LED fabrication

Figure S2(a) shows the schematic of the high-speed micro-LED. The devices were fabricated with a semi-transparent *p*-contact and an RF electrode design for ground-signal-ground probing. First, 200 nm of semi-transparent indium-tin-oxide (ITO) was blanket deposited on the structure using electron-beam evaporation to form the *p*-contact. Next, circular mesa structures were patterned on the ITO and dry etched in an inductively coupled plasma (ICP) system. Both the ITO and semiconductor layers were etched under the same conditions using Cl_2 plasma. The etch rates were determined to be 30 nm/min and 265 nm/min for the ITO and semiconductor layers, respectively. The ITO was subsequently annealed in a rapid thermal annealer (RTA) at 550 ^{o}C for

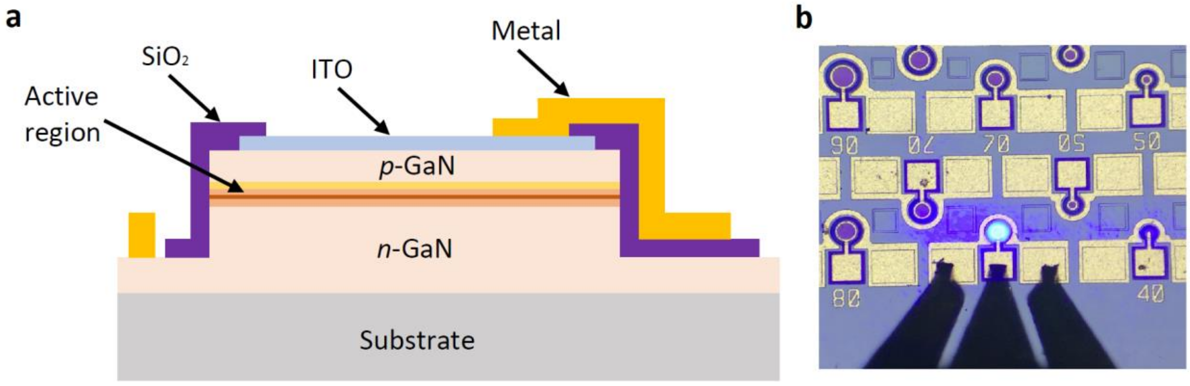

Figure S2 (a) Schematic of the high-speed micro-LED. (b) Top view optical microscope image of an LED under RF micro-probe.

10 minutes in N_2 to enhance transparency and reduce the resistivity. 175 nm of SiO_2 was deposited

to provide dielectric isolation between the *p*-side and *n*-side of the devices. Ti/Al/Ni/Au (20/100/50/50 nm) and Cr/Au (20/850 nm) were deposited as the *n*-contact and *p*-contact pads, respectively. Fig. S2(b) shows the top view optical microscope image of an LED under RF micro-probe. The electroluminescence spectrum of the LED at a current density of 1 kA/cm^2 is shown in Fig. S3. The peak wavelength is 435 nm.

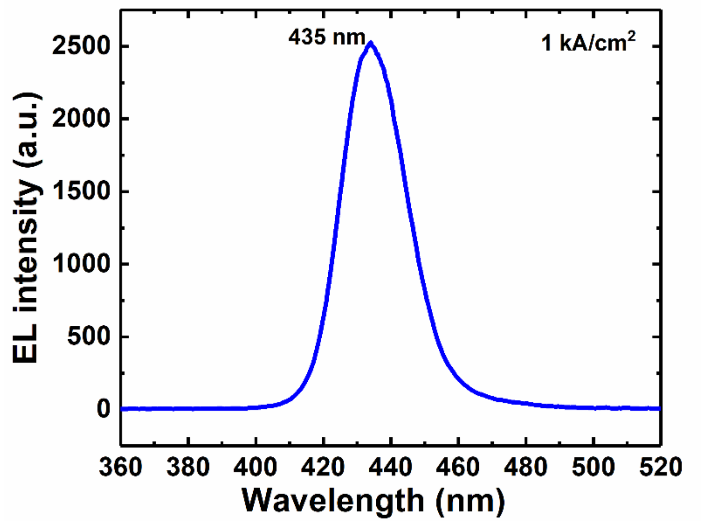

Figure S3 Electroluminescence spectrum of the InGaN/GaN LED at a current density of 1 kA/cm^2.

3. Calculation of the injection efficiency and carrier density

To calculate the injection efficiency, Eq. (1) and (2) are considered in steady-state form. The injection efficiency is the current associated with recombination in the quantum-well (QW) ($I_w = \frac{qN_w}{\tau_{rec}}$) divided by the total current injected into the device. Therefore, the total injection efficiency is

$$\eta_{inj} = \frac{I_W}{I} = \frac{\frac{qN_w}{\tau_{rec}}}{I} = \frac{1}{1 + \frac{\tau_c}{\tau_{rec,clad}}\left(1 + \frac{\tau_{rec}}{\tau_{esc}}\right)} \tag{S1}$$

However, since the small-signal measurement yields the differential carrier lifetimes, the differential forms of Eq. (1) and (2) should be used instead to find the injection efficiency. The differential forms of Eq. (1) and (2) are:

$$\frac{d}{dt}(dN_c) = \frac{dI}{q} - \frac{C_{sc}}{q}\frac{d}{dt}(dV_c) - \frac{dN_c}{\tau_{\Delta c}} + \frac{dN_w}{\tau_{\Delta esc}} - \frac{dN_c}{\tau_{\Delta rec,clad}} \tag{S2}$$

$$\frac{d}{dt}(dN_w) = \frac{dN_c}{\tau_{\Delta c}} - \frac{dN_w}{\tau_{\Delta rec}} - \frac{dN_w}{\tau_{\Delta esc}} \tag{S3}$$

By direct analogy to Eq. (S1), and using Eq. (S2) and (S3) in steady-state, the differential injection efficiency is derived as

$$\eta_{\Delta inj} = \frac{dI_w}{dI} = \frac{q\frac{dN_w}{\tau_{\Delta rec}}}{dI} = \frac{1}{1 + \frac{\tau_{\Delta c}}{\tau_{\Delta rec,clad}}(1 + \frac{\tau_{\Delta rec}}{\tau_{\Delta esc}})} \tag{S4}$$

The differential and total injection efficiencies are related through Eq. (S5).

$$\eta_{inj} = \frac{I_w}{I} = \frac{\int_0^I \eta_{\Delta inj} dI}{I} \tag{S5}$$

The extracted lifetimes from the fittings yield the total and differential injection efficiencies.

To calculate the total carrier number in the QW, Eq. (S2) and (S3) are considered in steady-state. Solving Eq. (S2) and (S3) for N_w leads to:

$$N_w = \frac{1}{q}\int \eta_{\Delta inj}\tau_{\Delta rec} dI \tag{S6}$$

The carrier density (n_w) is then calculated by knowing the total carrier number using $n_w = \frac{N_w}{dA}$, where d and A are the QW thickness and LED area, respectively.

4. Example of fitting

Equation (S7) and (S8) are the input impedance and modulation response of the circuit in Fig. 2(c).

$$Z_{in}(\omega) = R_s + \frac{R_{rec,clad}(R_w + R_c(1 + j\omega\tau_{\Delta rec}))}{(1 + j\omega\tau_{\Delta rec})\left(R_{rec,clad} + R_c(1 + j\omega\tau_{\Delta 0})\right) + R_w(1 + j\omega\tau_{\Delta 0})} \quad \text{(S7)}$$

$$H(\omega) = R_{rec,clad}R_w[R_s\left(R_{rec,clad}(1 + j\omega\tau_{\Delta rec}) + R_c(1 + j\omega\tau_{\Delta 0})(1 + j\omega\tau_{\Delta rec})\right.$$

$$\left. + R_w(1 + j\omega\tau_{\Delta 0})\right) + R_cR_{rec,clad}(1 + j\omega\tau_{\Delta rec}) + R_{rec,clad}R_w]^{-1} \quad \text{(S8)}$$

where $\tau_{\Delta 0} = R_{rec,clad}(C_c + C_{sc}) = \tau_{\Delta rec,clad} + R_{rec,clad}C_{sc}$, and $\tau_{\Delta rec} = R_wC_w$. Simultaneous fitting of the modulation response and input impedance are necessary to ensure unique solutions

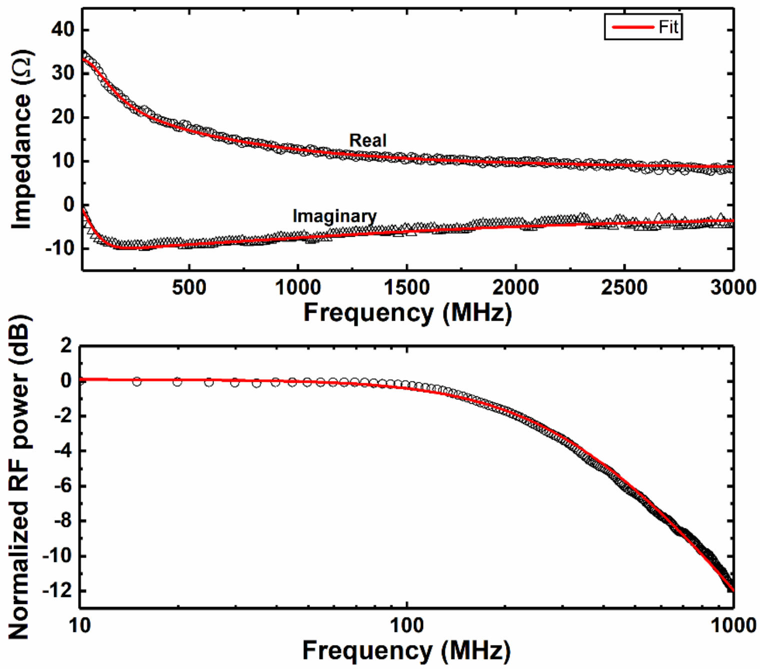

Figure S4 Simultaneous fitting to the measured data of expressions for real part of the impedance, imaginary part of the impedance, and 20log of the modulation response. Confidence intervals on the predicted data are very close to the predicted data, such that they are indistinguishable in the plots.

for the equations of (S7) and(S8). Figure S4 shows an example of simultaneous fitting to the measured data of expressions for real part of the impedance, imaginary part of the impedance, and 20log of the modulation response. Table S1 shows an example of the estimated fitting parameters and the 95% confidence intervals calculated for those parameters. All the estimated parameters fall within their confidence intervals, ensuring robustness of the fittings.

The impedance reduces with higher current densities, but in this case it is still relatively large due to the small area of the micro-LEDs, ensuring robust fittings as it is shown in Fig. S4 and Table S1.

Table S1: Estimated fitting parameters and their confidence intervals.

**Fitting parameter**	**Lower confidence boundary**	**Estimated parameter**	**Higher confidence boundary**
$\boldsymbol{R_c}$ $(\boldsymbol{\Omega})$	15.730	15.951	16.172
$\boldsymbol{R_w}(\boldsymbol{\Omega})$	9.088	9.464	9.840
$\boldsymbol{\tau_{rec}}$ $(\boldsymbol{s})$	7.425×10^{-10}	7.611×10^{-10}	7.797×10^{-10}
$\boldsymbol{\tau_{\Delta 0}}$ $(\boldsymbol{s})$	2.187×10^{-10}	2.249×10^{-10}	2.310×10^{-10}

5. Carrier escape time

Figure S5 shows the net carrier escape time as a function of current density for different stage temperatures. The carrier escape time initially decreases with increasing current density but starts to increase at high injection levels and eventually becomes negative around a current density of 3-4 kA/cm^2. The initial behavior of the carrier escape time is attributed to the carrier leakage from the QW. At high carrier injection levels, the population of carriers in the QW and cladding layers become similar, resulting in Coulomb-enhanced capture which leads to a negative escape rate. Carrier escape time generally reduces with increasing stage temperature due to increase of thermionic emission. The effect of carrier escape time is folded into the injection efficiency of Fig. 3(b).

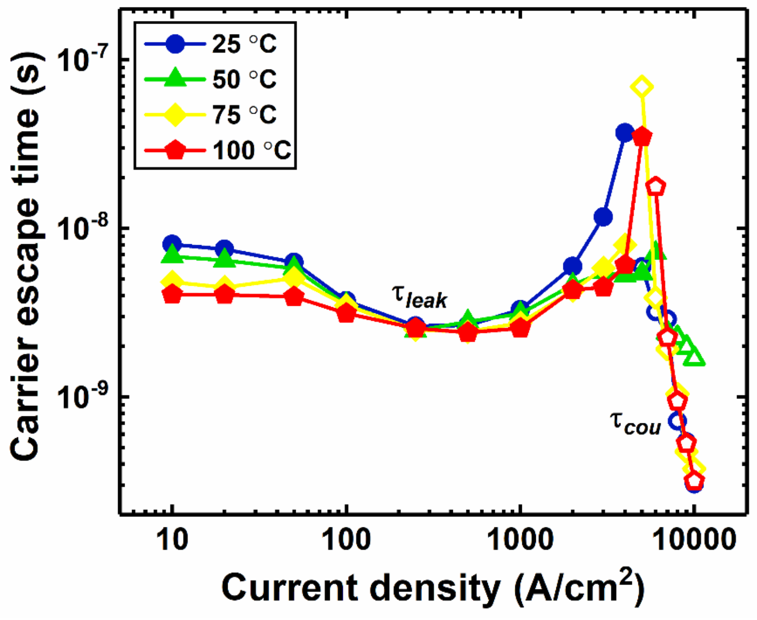

Figure S5 The net carrier escape time as a function of current density for different stage temperatures. Closed symbols when carrier escape time is positive ($\tau_{\Delta cou} > \tau_{\Delta leak}$) and open symbols when carrier escape time is negative ($\tau_{\Delta cou} < \tau_{\Delta leak}$).

6. Circuit parameters

The fitting procedure in section 4 resulted in the extraction of the parameters in equations S7 and S8. Figure S6 shows R_c, resistance associated with carriers in the cladding region, R_w, resistance associated with carriers in the QW, R_s, parasitic series resistance, $R_{rec,clad}$, resistance associated with carriers that recombine in the cladding region, C_c, capacitance associated with carriers in the cladding region, and C_w, capacitance associated with carriers in the QW as a function of current density. Detailed discussion of the behavior of the circuit parameters can be found in our previous works.[1,2]

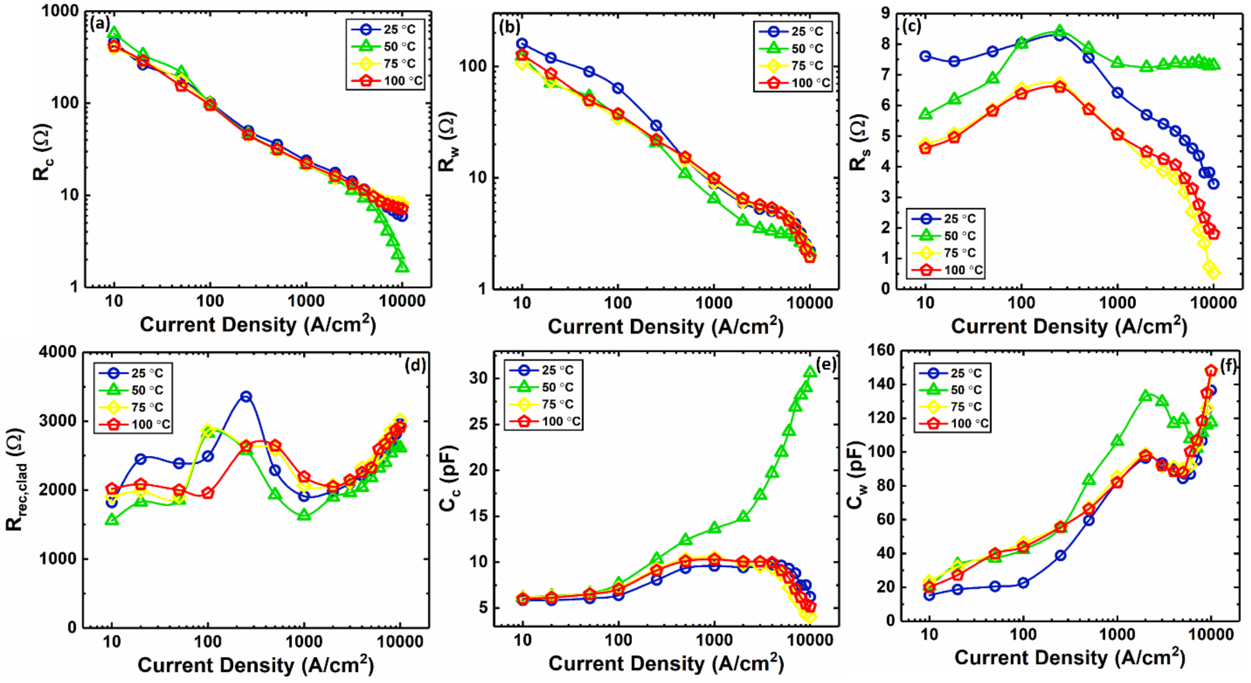

Figure S6 (a) R_c, resistance associated with carriers in the cladding region, (b) R_w, resistance associated with carriers in the QW, (c) R_s, parasitic series resistance, (d) $R_{rec,clad}$, resistance associated with carriers that recombine in the cladding region, and (e) C_c, capacitance associated with carriers in the cladding region, and (f) C_w, capacitance associated with carriers in the QW.

References

1 Rashidi, A., Monavarian, M., Aragon, A. & Feezell, D. Exclusion of injection efficiency as the primary cause of efficiency droop in semipolar (20 2¯ 1¯) InGaN/GaN light-emitting diodes. *Appl. Phys. Lett.* **113** (2018).

2 Rashidi, A. *et al.* Differential carrier lifetime and transport effects in electrically injected III-nitride light-emitting diodes. *J. Appl. Phys.* **122**, 035706 (2017).
